# The Utility of Peptide Ligand-Functionalized Liposomes for Subcutaneous Drug Delivery for Arthritis Therapy

**DOI:** 10.3390/ijms24086883

**Published:** 2023-04-07

**Authors:** Hemalatha Nanjaiah, Kamal D. Moudgil

**Affiliations:** 1 Department of Microbiology and Immunology, University of Maryland School of Medicine, Baltimore, MD 21201, USA; 2 Division of Rheumatology, Department of Medicine, University of Maryland School of Medicine, Baltimore, MD 21201, USA

**Keywords:** arthritis, adjuvant arthritis, rheumatoid arthritis, liposomes, nanotechnology, dexamethasone, peptide, surface-functionalization, targeted drug delivery

## Abstract

Liposomes and other types of nanoparticles are increasingly being explored for drug delivery in a variety of diseases. There is an impetus in the field to exploit different types of ligands to functionalize nanoparticles to guide them to the diseased site. Most of this work has been conducted in the cancer field, with relatively much less information from autoimmune diseases, such as rheumatoid arthritis (RA). Furthermore, in RA, many drugs are self-administered by patients subcutaneously (SC). In this context, we have examined the attributes of liposomes functionalized with a novel joint-homing peptide (denoted ART-1) for arthritis therapy using the SC route. This peptide was previously identified following phage peptide library screening in the rat adjuvant arthritis (AA) model. Our results show a distinct effect of this peptide ligand on increasing the zeta potential of liposomes. Furthermore, liposomes injected SC into arthritic rats showed preferential homing to arthritic joints, following a migration profile in vivo similar to that of intravenously injected liposomes, except for a less steep decline after the peak. Finally, liposomal dexamethasone administered SC was more effective than the unpackaged (free) drug in suppressing arthritis progression in rats. We suggest that with suitable modifications, this SC liposomal treatment modality can be adapted for human RA therapy.

## 1. Introduction

Nanotechnology has revolutionized the conceptual and practical framework underlying drug delivery and the pharmacological and pharmaceutical aspects of drug therapy [1,2]. Liposomes and a variety of other nanoparticles are being explored as carriers of drugs for the treatment of several disorders, particularly cancer [3,4]. The entrapment of drugs inside nanoparticles is shown to enhance the half-life of the drug, to increase the bioavailability and delivery of poorly soluble drugs, and/or to reduce the dosing frequency, which collectively lead to an improved therapeutic profile of that drug [4,5,6,7,8]. A critical advance in nanoparticle-based drug delivery is the realization that surface functionalization of such nanocarriers with certain ligands (e.g., antibodies, peptides, etc.) can aid in guiding them preferentially to the diseased site [9,10,11]. This tissue/organ-targeted drug delivery can further improve the therapeutic profile of that drug. Most of the above-mentioned attributes of nanotechnology have been examined in cancer, with relatively limited information obtained from the study of autoimmune diseases, such as rheumatoid arthritis (RA). Accordingly, knowledge gained from the former field can benefit the development of novel therapeutic modalities for RA and other autoimmune diseases.

RA is a chronic inflammatory disease affecting the synovial joints [12], which, if not treated adequately, may lead to deformity and/or disability. Within the joints, the synovial tissue is the site of immune cell infiltration and fibroblast proliferation [12,13]. However, the systemically-administered anti-arthritis drugs are distributed all over the body, affecting several other organs besides the joints, thereby increasing the risk of unwanted adverse effects of treatment [14,15]. In this regard, the targeted drug delivery approaches being developed for cancer are also of relevance for arthritis therapy. For that, there is a need to identify joint-homing ligands that can be used for the surface-functionalization of drug-entrapping nanoparticles. A few such potential ligands have indeed been identified by others [16,17,18] and us [19,20], but they have not yet been fully exploited for joint-targeted drug delivery in a form suitable for translation to human RA. Furthermore, considering that RA patients self-administer subcutaneously (SC) some of the commonly used anti-arthritis drugs [21,22], it is imperative to demonstrate that any functionalized nanoparticles are usable and effective in suppressing arthritis progression when given via the SC route. In addition, the relative outcomes in terms of reduced disease severity for nanoparticle-based SC treatment versus the unpackaged (free) drug SC treatment need to be determined.

Using the rat adjuvant arthritis (AA) model, we describe here a peptide-targeted liposomal SC drug delivery modality that meets both these requirements. We employed a novel joint-homing peptide ART-1, which was identified earlier following phage peptide library screening in arthritic (AA) rats [19,20]. We showed that intravenous injection of liposomes displaying this peptide and entrapping an experimental biologic, interleukin-27 (IL-27), was more effective in inhibiting arthritis than IL-27-entrapping liposomes lacking this peptide [20]. The current study extends and reinforces the outcomes of the above study [20] but uses another drug (dexamethasone; Dex) and a different route of delivery, namely SC. These validations are essential to de-risk the eventual translation of our peptide-guided drug delivery technology from the pre-clinical rat model (AA) to human RA.

## 2. Results and Discussion

### 2.1. Characterization of ART-1-Functionalized Liposomes Entrapping Cyanine 7 (Cy7) or Dexamethasone (Dex)

We examined the characteristics of a panel of liposomes to assess the impact of entrapment of Cy7 (a near-infrared dye useful for live imaging of rats) or Dex (a mainstream anti-arthritis drug) within them as well as their surface-functionalization with ART-1 peptide (as a lipopeptide with Octadecanamine). Liposomes that both lacked any cargo as well as surface ART-1 served as the baseline (Liposome-control) (Table 1). As expected, the entrapment of Cy7 or Dex in ART-1-liposomes resulted in a significant (*p* < 0.05) increase in the size of liposomes compared to Liposome-control. We then evaluated the effect of ART-1 on zeta potential (ZP), which represents an overall charge of the liposomes. ZP induces repulsion of nanoparticles, which is necessary to prevent their aggregation and, thereby, to improve their physical stability in vivo/in vitro [4,23]. Interestingly, the incorporation of ART-1 into the liposomal surface had a marked effect on ZP. The ZP of ART-1-liposomes, regardless of the presence or absence of Cy7/Dex, was positive in charge and much higher in magnitude, whereas that of liposomes lacking ART-1 (Liposome-control and Liposome-Dex) was negative in charge and much lower in quantity (Table 1). The above-mentioned change in ZP induced by ART-1-functionalization, along with the presence of polyethylene glycol (PEG) on the liposomal surface, might be of relevance in facilitating the dispersion of liposomes in vivo in the blood, body fluids, and tissues [4,23]. We speculate that this added benefit of ART-1, besides its joint-homing attribute, would be an asset for the use of these liposomes for arthritis therapy.

### 2.2. Live Imaging of Arthritic and Control Rats at Different Time Points after Injection of Cy7-Liposomes SC or IV

**Figure 1:**
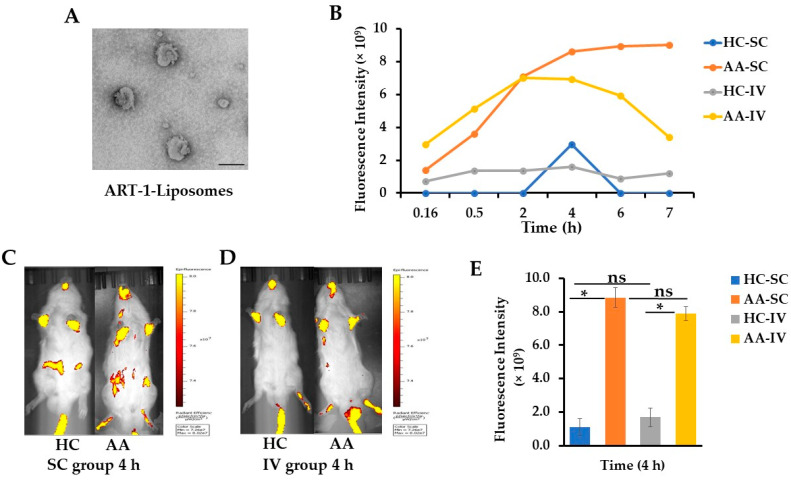

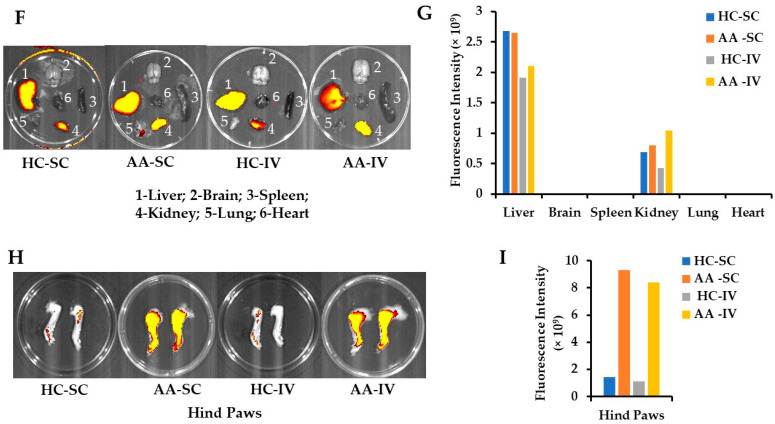
In vivo distribution of Cy7-entrapping ART-1-displaying liposomes in arthritic rats. (**A**) TEM image of ART-1-liposomes. The scale on the bottom right side is 100 nm; (**B**) Time kinetics of live imaging at the indicated time points in healthy representative control (HC) and adjuvant arthritis (AA) rats after subcutaneous (SC) or intravenous (IV) injection of Cy7-containing ART-1-liposomes; (**C**–**E**) Live imaging of control and arthritic rats (*n* = 3 per group each) at 4 h after SC/IV injection of liposomes. Values shown in sub-figure **E** are mean ± SEM; (* = *p*< 0.05); (**F**–**I**) Ex vivo imaging of various internal organs (**F**–**G**) and hind paws (**H**–**I**) harvested from representative control and arthritic rats, whose live imaging is shown in sub-figures above (**C**,**D**).

Using peptide ART-1-displaying liposomes (Figure 1A) entrapping Cy7 dye, we examined their biodistribution following SC injection into rats with adjuvant arthritis (AA) and compared the pattern with that following IV injection as a reference (control). Our objective was to determine the relative kinetics of liposomal biodistribution following these two routes of administration. Each rat received 100 uL ART-1-Cy7 liposomes SC or IV. The time points (h) tested included: 0.16, 0.5, 2, 4, 6, and 7 h (Figure 1B). The fluorescence intensity in the two hind paws was used for comparison among different groups.

Following SC injection, after a period of gradual increase initially, the fluorescence intensity maintained a sustained level at 6 h and 7 h (Figure 1B). After IV injection, there was a gradual increase in fluorescence intensity until 4 h post-injection, followed by a gradual decrease by 7 h (Figure 1B). In each case, the signal was not detectable at 48 h (data not shown). A comparison of fluorescence intensity of hind paws at 4 h after SC/IV injection of Cy7-liposomes is shown in Figure 1C–E. Taken together, overall, the peak level of fluorescence via the SC route was comparable to that via the IV route in liposomal targeting of the arthritic joints of hind paws.

### 2.3. Ex Vivo Imaging of Various Internal Organs and Hind Paws Harvested at 4 h Post-Liposome Injection of AA and Control Rats

To gain more insight into the biodistribution of liposomes to other organs besides hind paws, we assessed how the systemically administered liposomal Cy7 given SC or IV was distributed among different internal organs, namely the liver, brain, spleen, kidney, lung, and heart (indicated as #1–6 in that sequence in Figure 1F–G) at 4 h time point post-injection of ART-1-Cy7-liposomes (100 uL/rat). For reference, we also imaged the corresponding excised hind paws of those rats (Figure 1H–I).

For both SC and IV groups, the fluorescence signal was detectable in the liver and kidney, which represent the known excretion routes of Cy7 [24], but not in other internal organs tested, namely the brain, spleen, lung, and heart. The overall pattern of biodistribution of liposomes was comparable for the two arthritic rat groups (SC and IV).

Furthermore, as expected, a high level of fluorescence signal was observed in the hind paws of AA rats but not in those of healthy control (HC) rats. However, for the internal organs, no difference was observed in the AA rats compared to healthy rats. A positive fluorescence signal in the liver and kidney is attributable to dye (Cy7) excretion through these two organs [24], and it is comparable in AA and healthy rats. Thus, arthritis pathology itself did not alter the biodistribution of liposomes in vivo. These results are of relevance in the context of off-target adverse effects of potential anti-arthritis drugs used for entrapment in ART-1 liposomes for arthritis therapy. These results suggest rather limited if any, likely off-target effects on other organs (besides the excretion route organs) that can be attributed solely to the biodistribution properties of ART-1-liposomes. In sum, the results of our study show that the SC route of liposomal administration is well-suited for use in targeted drug delivery of anti-arthritis agents.

### 2.4. The Use of SC-Administered Dex-Entrapping ART-1-Functionalized Liposomes for Arthritis Therapy in Rats

Considering that anti-arthritis drugs are frequently administered SC by RA patients at home, the SC route of Dex delivery might be more practical than the IV route; the latter requires a hospital/clinic facility [21,22]. In this context, we tested the efficacy of the SC route of delivery for Dex for arthritis suppression in rats with AA. Beginning at the onset of AA, a cohort of rats was randomized into sub-groups, and Dex was delivered SC either as liposomal Dex (entrapped in ART-1 liposomes) or as unpackaged (free) Dex in solution. Another group of AA rats given the same dose of Dex but IV served as a reference (control) group, whereas AA rats that did not receive Dex served as positive controls. A total of six injections were given to each group of these rats on alternate days.

**Figure 2:**
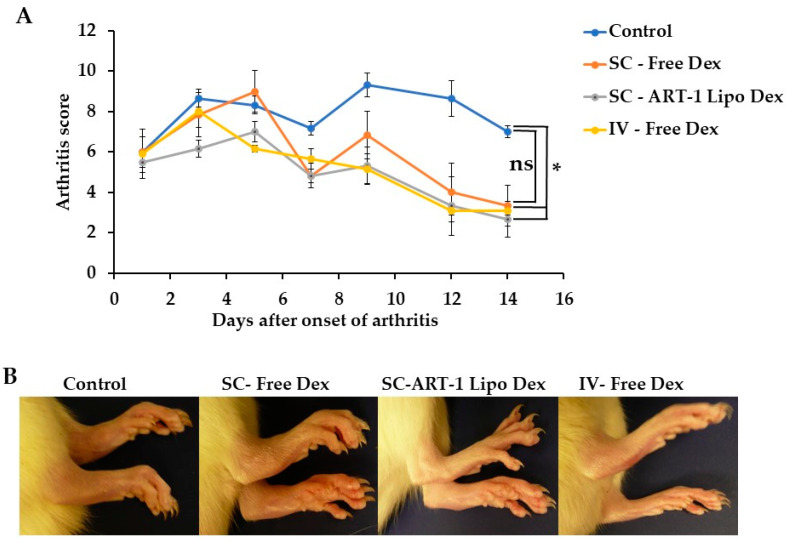
Determining the efficacy of liposomal Dex vs. unpackaged (free) Dex administered SC or IV. (**A**) Arthritis scores (mean ± SEM) of rats treated with the indicated preparations of Dex and untreated control rats. (* = *p* < 0.05; ns = not significant; using Wilcoxon rank sum test). (Abbreviations: SC—Subcutaneous, IV—Intravenous, Dex—Dexamethasone, ART-1-Lipo Dex—ART-1-Liposomes entrapping Dex, and Free Dex—unpackaged Dex). (**B**) Photographs of hind paws of representative rats, one from each group, whose Arthritis scores are shown in section A.

Our results (Figure 2) show that out of the three rat groups treated with Dex, each compared with the positive control group, arthritis suppression was most marked in the ART-1-liposomal-Dex SC group and the free Dex IV group. The difference in arthritis reduction in each of these groups was statistically significant (*p* < 0.05) when compared with the positive control group. In contrast, although the free Dex SC group showed a trend towards arthritis reduction, the difference from the positive control group was not significant (*p* > 0.05). Thus, the arthritis-suppressive ability of Dex was significantly improved when it was delivered in ART-1-liposomes than that in free (unpackaged) form. The former was comparable with free Dex given IV. These results are encouraging for the translational application of the ART-1-liposomal drug delivery via the SC route to RA patients. Furthermore, this study extends and reinforces the conclusions of our previous study in which ART-1-targeted liposomes were shown to deliver an experimental biologic (not yet approved for human use), interleukin-27 (IL-27), when injected IV into arthritic rats [20]. We now show here that the ART-1-functionalized liposomes entrapping a mainstream anti-arthritis drug, Dex, are also effective in suppressing arthritis when given SC (Figure 1 and Figure 2). The relevance of the SC route, as mentioned above, relates to RA patients self-administering (SC) commonly used anti-arthritic drugs.

## 3. Materials and Methods

### 3.1. Peptide-Functionalized Liposomes for Live Imaging and Arthritis Therapy

Liposome preparation was performed following the procedure of the thin film hydration method previously optimized in our laboratory [20], but with slight modifications, including the formulation of Dex used. A mixture of 4 lipids was used: DOPC (1,2-dioleoyl-sn-glycero-3-phosphocholine), DOPE (1,2-dioleoyl-sn-glycero-3-phosphoethanolamine), cholesterol, and DSPE-PEG (2000) amine (1,2-distearoyl-sn-glycero-3-phosphoethanolamine- Polyethylene glycol (2000) amine) (Sigma Aldrich, St. Louis, MO, USA, and Avanti polar lipids, Alabaster, AL, USA). The peptide ligand ART-1 was used for surface-functionalization (of liposomes) in the form of a lipopeptide, where an Octadecanamine tail was attached to the peptide portion (Lifetein, Somerset, NJ, USA) [20]. This lipopeptide, along with dexamethasone (Dex; D4902) (Sigma, USA) or Cyanine 7 (Cy7) (Lumiprobe, Hunt Valley, MD, USA), was added to the above mixture of 4 lipids. The entire mixture was then dried using nitrogen gas, followed by a hydration step and sequential sonication. Unencapsulated Dex was removed from the liposome suspension by centrifuge-filtration. The retentate containing liposomes was collected. During the optimization phase, these liposomes were examined by transmission electron microscopy (TEM) for shape and size. Thereafter, all liposomes were tested by a Zetasizer (DLS Malvern Zeta sizer, Nano) for their size (nm), polydispersity index (PDI), and Zeta potential (ZP) [20]. Furthermore, the entrapment of Dex within liposomes was measured by lysis of liposomes using Triton-X-100, and the released Dex was quantified using high-performance liquid chromatography (HPLC) (Waters Inc., photodiode detector, Milford, MA, USA) using a C18 column.

### 3.2. Animal Model of Arthritis

Adjuvant arthritis (AA) was induced in 5–6 week old male Lewis rats by SC immunization with heat-killed *M. tuberculosis* H37Ra (Mtb) (Difco Laboratories) [25]. Thereafter, rats were observed regularly for signs of arthritis. The severity of arthritis was graded on a scale of 0 to 4 per paw, as described elsewhere [25]. Age- and sex-matched healthy Lewis rats were used as controls. All animal work was performed as per the guidelines of the UMB Institutional Animal Care and Use Committee (IACUC).

### 3.3. Live Imaging of Rats and Ex Vivo Imaging of Harvested Organs and Hind Paws

After the onset of AA (which appears around d 10 after Mtb injection), rats were injected SC or IV with 100 uL/rat of Cy-7-loaded ART-1 liposomes. This volume was selected after pilot testing of 3 different concentrations of Cy7-liposomes. A separate group of naïve (healthy) rats treated in the same manner served as controls. All these rats were then subjected to live imaging under anesthesia using IVIS (Xenogen) equipment (Perkin Elmer) [20]. Images were acquired at different time points post-injection. Readings from naïve rats provided the level of background fluorescence to consider when making conclusions for the AA rats. Furthermore, the harvested organs and hind paws were also subjected to imaging ex vivo. The fluorescence intensity of all images was quantified (total radiant efficiency, ROI) using the IVIS software, and the data were subjected to statistical analysis.

### 3.4. Treatment of Arthritic Rats Using Liposomal/Free Dex

After preliminary screening using different doses of Dex, a dose of 0.1 mg/Kg was selected for further testing. A cohort of Lewis rats was injected SC with Mtb for arthritis (AA) induction. At the time of onset of AA (about d 10 after Mtb injection), rats were randomized into four groups. Three of these groups were treated SC separately with ART-1-liposomal Dex, unpackaged (free) Dex, or received no Dex. Another group of rats given free Dex IV served as a reference. In each case, a total of 6 injections (0.1 mg/Kg Dex each) were given every other day, starting from the day of onset. Thereafter, rats were graded regularly for the severity of arthritis following standard grading criteria for each paw [25].

## 4. Conclusions

From our results described above, we concluded that: (a) the surface-functionalization of liposomes with ART-1 peptide resulted in a marked increase in their Zeta potential, which was altered in a desired direction to facilitate dispersion of liposomes in vivo; (b) overall, the time kinetics and quantitative aspects of the in vivo biodistribution to the joints of the liposomes administered SC were comparable to that of liposomes injected IV; and (c) Liposomal Dex given SC was highly effective in controlling arthritis progression, and its effect was superior to that of free Dex SC but comparable to that of free Dex IV. When taken together, the SC route is well-suited for use in targeted drug delivery of anti-arthritis agents using peptide ligand-functionalized liposomes for RA therapy.

## Figures and Tables

**Table 1:** Characteristics of liposomes.

	Liposome Preparation	Size (nm) (Mean ± SEM)	PDI (Mean ± SEM)	ZP (mV) (Mean ± SEM)
1	Liposome-control	113.5 ± 3.03	0.2 ± 0.01	−3.4 ± 2.42
2	Liposome-Dex	98.9 ± 1.08	0.3 ± 0.26	−6.0 ± 2.34
3	ART-1-Liposome-control	123.7 ± 3.88	0.2 ± 0.01	22.6 ± 1.33 *
4	ART-1-Liposome-Cy7	147.7 ± 2.44	0.3 ± 0.02	19.3 ± 0.46 *
5	ART-1-Liposome-Dex	160.2 ± 4.46	0.3 ± 0.02	26.9 ± 1.85 *

* Zeta potential (ZP) of all three ART-1-containing liposomes was significantly (*p* < 0.05) higher than that of liposomes lacking ART-1. However, the ZP of Liposome-control and Liposome-Dex was comparable (*p* > 0.05). (Abbreviations: Dex = dexamethasone; Cy7 = cyanine 7; PDI = polydispersity index.)

## Data Availability

Not applicable.

## References

[1] Heath J.R. (2015). Nanotechnologies for biomedical science and translational medicine. Proc. Natl. Acad. Sci. USA.

[2] Contera S., Bernardino de la Serna J., Tetley T.D. (2020). Biotechnology, nanotechnology and medicine. Emerg. Top. Life Sci..

[3] do Nascimento T., Todeschini A.R., Santos-Oliveira R., de Souza de Bustamante Monteiro M.S., de Souza V.T., Ricci-Júnior E. (2020). Trends in Nanomedicines for Cancer Treatment. Curr. Pharm. Des..

[4] Bozzuto G., Molinari A. (2015). Liposomes as nanomedical devices. Int. J. Nanomed..

[5] Devalapally H., Chakilam A., Amiji M.M. (2007). Role of nanotechnology in pharmaceutical product development. J. Pharm. Sci..

[6] Gokce E.H., Ozyazici M., Souto E.B. (2010). Nanoparticulate strategies for effective delivery of poorly soluble therapeutics. Ther. Deliv..

[7] Onoue S., Yamada S., Chan H.K. (2014). Nanodrugs: Pharmacokinetics and safety. Int. J. Nanomed..

[8] Bilia A.R., Piazzini V., Risaliti L., Vanti G., Casamonti M., Wang M., Bergonzi M.C. (2019). Nanocarriers: A Successful Tool to Increase Solubility, Stability and Optimise Bioefficacy of Natural Constituents. Curr. Med. Chem..

[9] Abd Ellah N.H., Abouelmagd S.A. (2017). Surface functionalization of polymeric nanoparticles for tumor drug delivery: Approaches and challenges. Expert Opin. Drug Deliv..

[10] Berillo D., Yeskendir A., Zharkinbekov Z. (2021). Peptide-Based Drug Delivery Systems. Medicina.

[11] Säälik P., Lingasamy P., Toome K., Mastandrea I., Rousso-Noori L., Tobi A., Simón-Gracia L., Hunt H., Paiste P., Kotamraju V.R. (2019). Peptide-guided nanoparticles for glioblastoma targeting. J. Control Release.

[12] Deane K.D. (2021). Rheumatoid Arthritis Pathogenesis, Prediction, and Prevention: An Emerging Paradigm Shift. Nat. Immunol..

[13] Jang S., Kwon E.J. (2022). Rheumatoid Arthritis: Pathogenic Roles of Diverse Immune Cells. Int. J. Mol. Sci..

[14] Cush J.J. (2022). Rheumatoid Arthritis: Early Diagnosis and Treatment. Rheum. Dis. Clin. N. Am..

[15] Radu A.F., Bungau S.G. (2021). Management of Rheumatoid Arthritis: An Overview. Cells.

[16] Lee L., Buckley C., Blades M.C., Panayi G., George A.J., Pitzalis C. (2002). Identification of synovium-specific homing peptides by in vivo phage display selection. Arthritis Rheum..

[17] Paulos C.M., Turk M.J., Breur G.J., Low P.S. (2004). Folate receptor-mediated targeting of therapeutic and imaging agents to activated macrophages in rheumatoid arthritis. Adv. Drug Deliv. Rev..

[18] Vanniasinghe A.S., Manolios N., Schibeci S., Lakhiani C., Kamali-Sarvestani E., Sharma R., Kumar V., Moghaddam M., Ali M., Bender V. (2014). Targeting fibroblast-like synovial cells at sites of inflammation with peptide targeted liposomes results in inhibition of experimental arthritis. Clin. Immunol..

[19] Yang Y.H., Rajaiah R., Ruoslahti E., Moudgil K.D. (2011). Peptides targeting inflamed synovial vasculature attenuate autoimmune arthritis. Proc. Natl. Acad. Sci. USA.

[20] Meka R.R., Venkatesha S.H., Moudgil K.D. (2018). Peptide-directed liposomal delivery improves the therapeutic index of an immunomodulatory cytokine in controlling autoimmune arthritis. J. Control Release.

[21] Bianchi G., Caporali R., Todoerti M., Mattana P. (2016). Methotrexate and Rheumatoid Arthritis: Current Evidence Regarding Subcutaneous Versus Oral Routes of Administration. Adv. Ther..

[22] Bittner B., Richter W., Schmidt J. (2018). Subcutaneous Administration of Biotherapeutics: An Overview of Current Challenges and Opportunities. BioDrugs Clin. Immunother. Biopharm. Gene Ther..

[23] Zhao W., Zhuang S., Qi X.R. (2011). Comparative study of the in vitro and in vivo characteristics of cationic and neutral liposomes. Int. J. Nanomed..

[24] Zou P., Xu S., Povoski S.P., Wang A., Johnson M.A., Martin E.W., Subramaniam V., Xu R., Sun D. (2009). Near-infrared fluorescence labeled anti-TAG-72 monoclonal antibodies for tumor imaging in colorectal cancer xenograft mice. Mol. Pharm..

[25] Moudgil K.D., Chang T.T., Eradat H., Chen A.M., Gupta R.S., Brahn E., Sercarz E.E. (1997). Diversification of T cell responses to carboxy-terminal determinants within the 65-kD heat-shock protein is involved in regulation of autoimmune arthritis. J. Exp. Med..

